# Contribution of Risk Factors, Including Polygenic Score, to the Multifactorial Risk Assessment for the Implementation of Personalized Breast Cancer Screening: Insights from the PERSPECTIVE: Integration and Implementation Project

**DOI:** 10.3390/cancers18091482

**Published:** 2026-05-05

**Authors:** Xin Yang, Juliet A. Usher-Smith, Kristina M. Blackmore, Jennifer D. Brooks, Kathleen A. Bell, Tim Carver, Amy Chang, Jocelyne Chiquette, Douglas F. Easton, Andrea Eisen, Laurence Eloy, Samantha Fienberg, Yann Joly, Raymond H. Kim, Bartha M. Knoppers, Laurence Lambert-Côté, Hermann Nabi, Nora Pashayan, Penny Soucy, Tracy L. Stockley, Annie Turgeon, Meghan J. Walker, Michael Wolfson, Michel Dorval, Anna M. Chiarelli, Antonis C. Antoniou, Jacques Simard

**Affiliations:** 1 Centre for Cancer Genetic Epidemiology, Department of Public Health and Primary Care, School of Clinical Medicine, University of Cambridge, Cambridge CB1 8RN, UKdfe20@medschl.cam.ac.uk (D.F.E.); np275@medschl.cam.ac.uk (N.P.); aca20@medschl.cam.ac.uk (A.C.A.); 2 Primary Care Unit, Department of Public Health and Primary Care, School of Clinical Medicine, University of Cambridge, Cambridge CB1 8RN, UK; jau20@medschl.cam.ac.uk; 3 Ontario Health, Toronto, ON M5G 2L3, Canada; kristina.blackmore@ontariohealth.ca (K.M.B.); kathleen.bell@ontariohealth.ca (K.A.B.); eisena@hhsc.ca (A.E.); raymond.kim@uhn.ca (R.H.K.); meghan.walker@ontariohealth.ca (M.J.W.); 4 Dalla Lana School of Public Health, University of Toronto, Toronto, ON M5S 1A1, Canada; jennifer.brooks@utoronto.ca (J.D.B.); anna.chiarelli@utoronto.ca (A.M.C.); 5 CHU de Québec-Université Laval Research Center, Quebec City, QC G1V 4G2, Canadahermann.nabi@crchudequebec.ulaval.ca (H.N.); penny.soucy@crchudequebec.ulaval.ca (P.S.); annie.turgeon@crchudequebec.ulaval.ca (A.T.); michel.dorval@crchudequebec.ulaval.ca (M.D.); 6 Department of Family Medicine and Emergency Medicine, Faculty of Medicine, Université Laval, Quebec City, QC G1V 0A6, Canada; 7 Centre for Cancer Genetic Epidemiology, Department of Oncology, University of Cambridge, Cambridge CB1 8RN, UK; 8 Sunnybrook Health Science Centre, Toronto, ON M4N 3M5, Canada; 9 Department of Oncology, McMaster University, Hamilton, ON L8V 5C2, Canada; 10 Programme Québécois de Cancérologie, Ministère de la Santé et des Services Sociaux, Quebec City, QC G1S 2M1, Canada; 11 Centre Intégré de Santé et de Services Sociaux de Lanaudière, Centre hospitalier de Lanaudière, Saint-Charles Borromée, QC J6E 6J2, Canada; 12 Centre of Genomics and Policy, McGill University, Montreal, QC H3A 0G1, Canada; yann.joly@mcgill.ca (Y.J.);; 13 Princess Margaret Cancer Centre, Toronto, ON M5G 2M9, Canada; tracy.stockley@uhn.ca; 14 Department of Social and Preventive Medicine, Faculty of Medicine, Université Laval, Quebec City, QC G1V 0A6, Canada; 15 Université Laval Cancer Research Center, Quebec City, QC G1R 3S3, Canada; 16 Division of Clinical Laboratory Genetics, University Health Network, Toronto, ON M5G 2C4, Canada; 17 Department of Laboratory Medicine and Pathobiology, University of Toronto, Toronto, ON M5S 1A8, Canada; 18 School of Epidemiology and Public Health, University of Ottawa, Ottawa, ON K1G 5Z3, Canada; 19 Faculty of Pharmacy, Université Laval, Quebec City, QC G1V 0A6, Canada; 20 Department of Molecular Medicine, Faculty of Medicine, Université Laval, Quebec City, QC G1V 4G2, Canada

**Keywords:** breast cancer risk stratification, polygenic score, risk prediction models, data collection for breast cancer screening program, implementation of multifactorial risk assessment

## Abstract

Background/Objectives: Risk-based breast cancer (BC) screening can provide tailored recommendations based on individual risk. We aimed to identify key predictors for BC risk stratification to inform implementation in screening programs. Methods: We estimated 10-year BC risks using BOADICEA v.6 (CanRisk) in 3753 women aged 40–70 with no cancer history from the PERSPECTIVE I&I cohort. The primary endpoint was risk reclassification, assessed as the proportion of women whose assigned 10-year risk category changed when using different risk factor combinations against a full multifactorial model including questionnaire-based risk factors (QRFs), polygenic score (PGS), mammographic density (MD), and pedigree-structured first- and second-degree family history (FH) of breast, ovarian, pancreatic and prostate cancer, including both affected and unaffected relatives. Relative risk thresholds were set as <1.5 (average), 1.5–2.7 (higher-than-average), and ≥2.7 (high), equivalent to the remaining lifetime risk categories of <15%, 15–25% and ≥25% for women aged 30 (the anchor) to age 80. We quantified individual-level reclassification flows by direction and magnitude. Results: Excluding PGS from risk calculations led to the highest overall reclassification. Using only the BC status in first- and second-degree relatives produced comparable risk classification to that of the full FH data that included breast, ovarian, prostate and pancreatic cancer (reclassification = 0.5%). However, collecting only affected relatives led to overestimation of risk. Excluding either PGS, MD or FH resulted in a greater proportion of reclassification among younger women. Adding the PGS to risk factors already collected in provincial screening programs reduced reclassification from 23% to ~13%. Conclusions: PGS, MD, QRFs and FH of BC in affected and unaffected first- and second-degree relatives are key for refining risk stratification. These findings provide real-world evidence on how incorporating different sets of risk factors, both those routinely collected in screening programs and those requiring additional data collection, affect individual-level risk classification amongst a population-based cohort, and how the impacts differ across age groups. While risk classification reflects model-based changes in estimated risk categories rather than direct evidence of mis-screening or clinical outcomes, comparison with the current eligibility criteria used to identify women at higher-than-average risk highlights the potential clinical value of a multifactorial risk assessment approach in ensuring more appropriate screening strategies.

## Simple Summary

Risk-based breast cancer screening offers the potential to tailor screening recommendations based on individual risk rather than age alone. Since the decision to offer earlier or more frequent screening is based on a pre-defined threshold, understanding reclassification at an individual level is important. This study uses real-world data from the PERSPECTIVE I&I cohort and the BOADICEA risk prediction model to evaluate how each major risk factor contributes to an individual woman’s risk category. Omitting polygenic score caused the greatest reclassification, particularly among women aged 40–49 years, similar to the effects observed for family history and mammographic density, while questionnaire-based factors had a greater influence in older women. Including family history of breast cancer only is sufficient, but including both affected and unaffected relatives is crucial to avoid risk overestimation. These findings highlight the importance of multifactorial risk assessment and provide practical evidence to streamline data collection and support scalable personalized screening strategies.

## 1. Introduction

Breast cancer (BC) screening programs currently use age as the primary criterion for eligibility. In Canada, women aged 50–74 are recommended for routine BC screening every two years [1,2], with some jurisdictions offering screening to women in their forties [3]. However, BC risk varies substantially between individuals, and age alone does not explain this variability. Sojourn time, reflecting the rate of progression during the preclinical screen-detectable phase, varies by BC subtype and is generally shorter for more aggressive tumors, but does not differ substantially by risk group. While higher risk is associated with earlier disease onset, it is not associated with faster progression [4]. Age-based screening fails to account for population heterogeneity in BC risk and disease dynamics, potentially leading to over- or under-screening, increased anxiety, and unnecessary costs [5,6,7,8]. Risk-based screening offers a more personalized approach by classifying individuals into different risk strata and tailoring screening recommendations accordingly. For example, women at higher risk may be offered earlier and more frequent screening to improve early detection and survival outcomes, whereas women at lower risk may initiate screening later, reducing unnecessary screening and associated harms. For example, in Ontario, Canada, annual mammography screening is recommended for women classified as “higher-than-average risk” based on first- and second-degree family history (FH) of breast and/or ovarian cancer or high breast density (“D” category in BI-RADS^®^ [9]) [10]. For women classified as “high-risk”—those with a FH of breast and ovarian cancer and an estimated lifetime risk of 25% or higher, or carriers of pathogenic variants in high-risk genes such as *BRCA1* or *BRCA2*—annual mammography and magnetic resonance imaging (MRI) is recommended [10]. However, these criteria rely on FH and genetic testing and, therefore, will likely fail to identify women at elevated risk who do not have an FH of cancer.

Recent evidence suggests that multifactorial risk assessment in the context of population screening programs could help better identify women at higher risk of BC. The implementation of such an approach would improve the benefit-to-harm ratio of screening and promote a more efficient use of already strained healthcare resources [11]. Given the substantial potential benefits of risk-based breast screening, many studies have focused on determining optimal approaches for its delivery, including evaluation of efficacy, effectiveness, cost-effectiveness, feasibility, acceptability, health system readiness, as well as social, ethical and legal issues related to risk prediction and communication, in order to facilitate implementation in the population-based screening programs [12,13,14,15,16,17,18,19,20,21].

Our research group has recently contributed to this international effort through the large-scale PERSPECTIVE I&I project (Personalized Risk Assessment for Prevention and Early Detection of Breast Cancer: Integration and Implementation), which aimed to improve BC risk assessment and identify optimal strategies for implementing risk-based screening and prevention in Canada [22]. As part of PERSPECTIVE I&I, a large prospective cohort study was conducted, which recruited eligible women from two provinces in Canada, namely Quebec and Ontario, to undergo a multifactorial BC risk assessment, including PGS, and receive screening and prevention recommendations according to their risk category [23]. The project generated the first Canadian evidence on multifactorial BC risk assessment in the population setting, specifically producing evidence on feasibility, acceptability, uptake, healthcare resource utilization, and socio-ethical and legal considerations, to inform and support the implementation of a personalized, risk-based approach within organized breast screening programs [23,24,25,26,27,28,29].

The multifactorial Breast and Ovarian Analysis of Disease Incidence and Carrier Estimation Algorithm (BOADICEA), implemented in the CanRisk tool (www.canrisk.org, accessed on 1 May 2026) [30], is one model that enables such multifactorial risk assessment. It incorporates a broad range of risk factors, including age, pedigree-structured cancer FH, rare high- or moderate-risk genetic susceptibility genes, common low-risk single-nucleotide polymorphisms (SNPs) summarized in polygenic scores (PGSs), a wide range of questionnaire-based risk factors (QRFs), including lifestyle, reproductive and hormone-related risk factors, and mammographic density (MD, in BI-RADS^®^ categories or continuous measures [31]), with different weights assigned to each risk factor [32]. Previous validation studies in prospective cohorts have shown that considering the full set of these risk factors provides the best-calibrated and most discriminative risk estimates in both population-based and screening cohorts [33,34,35], as well as in a clinical genetics setting for women carrying *BRCA1* or *BRCA2* pathogenic variants [36]. However, the collection of accurate information for all the risk factors considered in BOADICEA in practice remains a significant challenge and is resource-intensive [23]. The optimal approach to implementing multifactorial risk assessment into screening programs may, therefore, not include all known risk factors. While the impact of removing risk factors from multifactorial models on discrimination, calibration and overall risk classification has been reported in validation studies, the impact of removing groups of risk factors on individual-level classification of risk within screening programs is not known. Since the decision to offer earlier or more frequent screening is based on pre-defined thresholds, understanding reclassification at an individual level is important.

The current study uses data from the PERSPECTIVE I&I project to assess the specific contribution of individual BC risk factors to overall risk classification. The goal is to provide real-world evidence on the impact of varying sets of risk factors, both those already collected within screening programs and those that would require additional data collection, on the classification of risk amongst a population-based cohort, and how the impacts differ across age groups. The findings from our study will help rationalize data collection and maximize resource utilization within the screening programs, thereby guiding the development of optimal personalized screening strategies.

## 2. Materials and Methods

### 2.1. Datasets

Details of the PERSPECTIVE I&I study design and participant recruitment have been described elsewhere [22,23]. Briefly, women aged 40–70, unaffected by BC and who had a previous mammogram, were recruited to participate in the prospective cohort study from July 2019 to December 2021. Participants were recruited from the two most populous Canadian provinces, namely Ontario and Quebec. Recruitment methods differed between the two provinces to reflect their distinct clinical and operational policies. In Ontario, women aged 50–69 who had undergone a mammogram at one of the Ontario Breast Screening Program (OBSP) sites received invitation letters. Additional participants aged 40–69 were recruited through advertisements in mammography centers, primary care clinics, websites, newsletters, and social media. In Quebec, recruitment relied on advertisements in mammography centers, traditional and social media, email listservs of partner organizations, and a study website. To participate, women had to have a primary care provider and have previously undergone a mammogram at a screening center in selected regions. Women were eligible for inclusion if they had not already been identified as high-risk, had no personal history of cancer, no mastectomy and had not undergone genetic testing or counseling for BC. Additional details regarding participant recruitment and selection are provided in [23]. A total of 3753 participants, 2111 from Ontario and 1642 from Quebec, were included in the analyses described in the current study. A summary of participant characteristics is provided in Table A1. Informed consent was obtained from all subjects involved in the study.

Recruited participants completed questionnaires to collect FH and risk factor information, provided a saliva sample to determine the PGS, and consented to the collection of their most recent mammogram report to obtain MD, to facilitate the multifactorial risk assessment.

Specifically, lifestyle, reproductive and hormone-related risk factors were collected through self-completed questionnaires at recruitment, hereby referred to as QRFs. These included height, weight, parity, first live childbirth, menarche and menopause, oral contraceptive use, menopausal hormone therapy (MHT) use and alcohol intake [32] (Table A1). Self-reported FH of breast, ovarian, prostate and pancreatic cancers was also collected in pedigree format, including both affected and unaffected relatives, up to second-degree relatives.

Participants were asked to provide a saliva sample using a collection kit (DNA Genotek, Ottawa, ON, Canada) as a source of DNA for a clinical-grade Breast Cancer Genetic Risk SNP test. DNA was extracted from participants’ saliva samples collected at recruitment. A total of 295 SNPs out of the 313-SNP BC PGS [37,38] were genotyped and standardized as described previously [23]. Participants were not screened for pathogenic variants in monogenic cancer susceptibility genes (e.g., *BRCA1, BRCA2, PALB2, CHEK2,* and *ATM*). Mammogram reports were obtained from the electronic hospital records in Ontario and from participating screening centers in Quebec. MD was abstracted from mammogram reports and was recorded in BI-RADS^®^ categories [23].

Following data collection, eligible women underwent risk assessment using the multifactorial risk prediction BOADICEA tool to determine their risk category, after which they received a personalized screening and prevention plan aligned with their assigned category [23].

### 2.2. Breast Cancer Risk Prediction

Ten-year BC risks were predicted using BOADICEA v.6 [32] with age- and calendar period-specific Canadian population BC incidence rates. To assess the impact of individual risk factors on risk classification, we excluded each of the following, QRFs, PGS, MD and FH from the full model that includes QRFs, PGS, MD, and pedigree-structured first- and second-degree FH of breast, ovarian, pancreatic and prostate cancer, including both affected and unaffected relatives, and compared the resulting risk estimates to those generated using the full model. Analyses were conducted for the overall cohort and stratified by age groups 40–49 and 50–70 years to assess whether specific risk factors have a greater influence on risk classification in particular age groups. Within QRFs, hormonal and lifestyle factors were assessed separately. For FH, we further investigated the influence of the degree of relatives included, the types of cancer considered, and the impact of including unaffected relatives.

We also examined the risk classification based on the set of risk factors that were collected by the Ontario Breast Screening Program (OBSP) and the Programme québécois de dépistage du cancer du sein (PQDCS) (Table A2). We then assessed the improvement in risk classification by adding PGS to the OBSP and PQDCS risk factors.

The risk classification using the annual mammography screening recommendation criteria in the OBSP—which include mammographic density classified as category D in the Breast Imaging Reporting and Data System (BIRADS), as well as any of the following: two or more first-degree female relatives with BC at any age; one or more first-degree female relatives diagnosed with BC before the age of 50; one or more first or second-degree male relatives diagnosed with BC at any age; or one or more first-degree relatives diagnosed with ovarian cancer at any age—was compared with the risk assessment results using the full model. Since the PQDCS does not have such specific mammography screening recommendation criteria, we applied Ontario’s criteria to the entire dataset across both provinces.

BOADICEA also considers the effects of rare pathogenic variants in eight high- or moderate-risk BC susceptibility genes. However, these genes were not tested in the participants and were treated as unknown in the risk prediction for all analyses. For unknown or missing data, BOADICEA uses the average population effects across the relevant risk factor categories [32].

### 2.3. Statistical Analysis

We classified BC risks into three risk categories based on age-dependent risk thresholds using relative risk (RR), which was calculated as the ratio of the predicted 10-year absolute BC risks to the population age-specific average absolute risk. RR thresholds were set as <1.5 (average), 1.5–2.7 (higher-than-average), and ≥2.7 (high), equivalent to the remaining lifetime risk categories of <15%, 15–25% and ≥25% for women aged 30 (the anchor) to age 80 based on recommendations in [39].

To visualize the impact of different sets of risk factors on risk classification, we used Sankey plots to illustrate the proportion of individuals shifting between risk categories across models. The reclassification rate was calculated as the proportion of individuals moved to a lower or higher risk category compared to the full model. Statistical significance of differences in the distribution of risk categories between models was assessed by a chi-square test. All statistical analyses were performed in R (version 4.3.3).

## 3. Results

**Figure 1:**
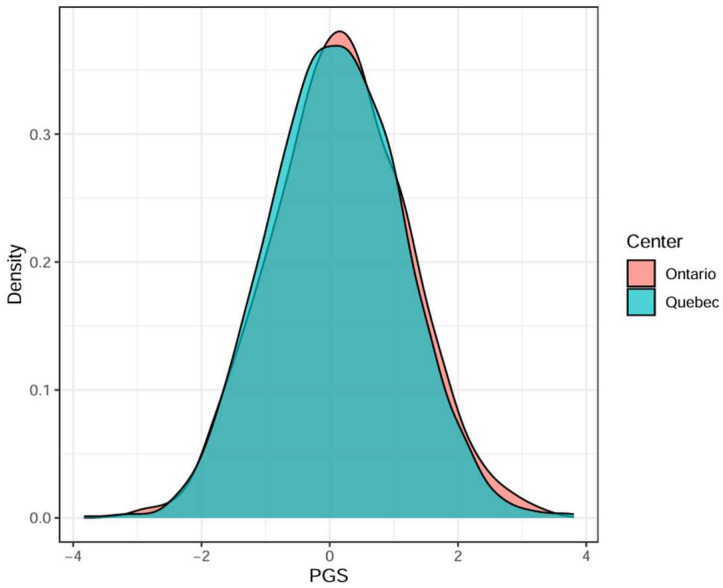
Comparison of PGS distribution between Ontario and Quebec study participants. The dataset included 2111 participants from Ontario and 1642 participants from Quebec.

A total of 3753 women aged 40–70 were included in the analysis, including 2111 (56.2%) from Ontario and 1642 (43.8%) from Quebec. Cohort characteristics are detailed in Table A1. The distribution of PGS was well aligned with the reference European population [23], with a mean of 0.138 and a standard deviation of 1.045 (Table A1), and did not differ significantly between the two provinces (*t*-test *p*-value = 0.07, Figure 1). Using the full model that included all risk factors, 78.2% of women were classified as average risk, 16.7% as higher-than-average risk and 5.2% as high risk.

### 3.1. Risk Reclassification Compared to the Full Model When Omitting Different Risk Factors

**Figure 2:**
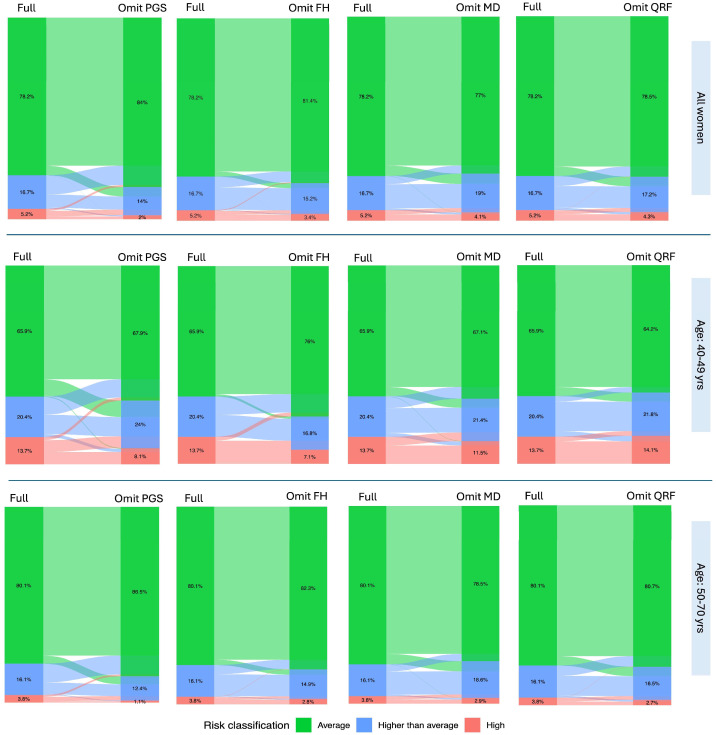
Risk reclassification when omitting the four major risk factor categories from the full model: polygenic risk score (PGS), family history (FH), mammographic density (MD) and questionnaire-based risk factors (QRFs) in all women (**top panel**), women aged 40–49 years (**middle panel**), and women aged 50–70 years (**bottom panel**). The flows illustrate transitions in individual risk classification from the full model (**left**) to the reduced model with specified risk factors omitted (**right**), with the width of each flow representing the proportion of individuals reclassified between risk categories.

Among the four major risk factor categories (QRFs, PGS, MD and FH), omitting PGS led to the highest level of reclassification (18.6%, *p* = 1.5 × 10^−15^), including 28.5% of reclassified women shifted to a higher risk category and 71.5% to a lower risk category. Excluding FH resulted in a reclassification of 10.3% (*p* = 9.0 × 10^−5^), with 28.2% of reclassified women moved to a higher risk category and 71.8% to a lower risk category. When MD was omitted, 12.1% of women were reclassified (*p* = 0.004), with an equal proportion moving up and down in risk category. Omitting QRFs led to 12.8% of women reclassified (*p* = 0.16), including 45.3% reclassified to a higher risk category and 54.7% to a lower risk category (Table 1A and Figure 2 top panel).

### 3.2. Risk Reclassification by Age

Of the cohort, there were 505 women (13.5%) aged 40–49 years, almost exclusively from Quebec, and 3248 women (86.5%) aged 50–70 years. Compared to the full model, excluding either PGS, MD, or FH resulted in a greater proportion of risk reclassification among younger women than older women (Table 1B,C and Figure 2 middle and bottom panel). In contrast, excluding QRFs led to more risk reclassification among older women. Notably, in the younger age group, omitting FH led to 93% of reclassified women shifted to a lower risk category, indicating substantial risk underestimation when FH is not considered in this age group (Table 1B). In comparison, when PGS or MD was omitted, 61.7% and 60.5% of reclassified women were assigned to a lower risk category, respectively.

### 3.3. Impact of Family History Information on Risk Classification

**Figure 3:**
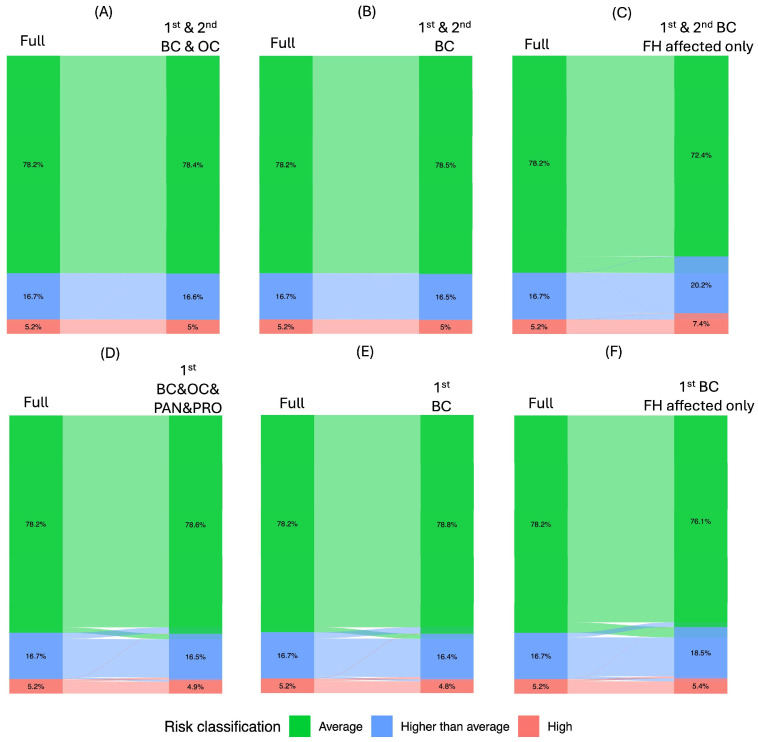
Risk reclassification when reducing the cancer types or/and degrees of relatives considered in the full model. (**A**) Full model (considering breast, ovarian, pancreatic and prostate cancers in the first- and second-degree relatives) versus just considering breast and ovarian cancer in the first- and second-degree relatives; (**B**) full model versus just considering breast cancer in the first- and second-degree relatives; (**C**) full model versus just considering breast cancer in the first- and second-degree relatives without considering unaffected relatives; (**D**) full model versus considering breast, ovarian, pancreatic and prostate cancers in the first-degree relatives only; (**E**) full model versus considering breast cancer in the first-degree relatives only; (**F**) full model versus considering breast cancer in the first-degree relatives only without considering unaffected relatives. The flows illustrate transitions in individual risk classification from the full model (**left**) to the reduced model with specified risk factors omitted (**right**), with the width of each flow representing the proportion of individuals reclassified between risk categories.

When compared to the full model which included all pedigree FH information, reducing the cancer types considered in first- and second-degree relatives from breast, ovarian, pancreatic, and prostate cancer (full) to just breast and ovarian, and further to BC only, resulted in minimal changes in risk classification (*p* = 0.96 and 0.90, reclassification = 0.3% and 0.5% respectively; Table 2, Figure 3A,B). However, when FH was further limited to affected relatives only, predicted risks significantly increased (*p* = 1.8 × 10^−8^), with 8.2% of women reclassified to a higher risk category versus 0.2% of women reclassified to a lower risk category (Figure 3C). Restricting FH to include all cancers exclusively in first-degree relatives led to 5.7% reclassification, which was similar to the results obtained when FH was limited only to BC in first-degree relatives (5.6%) (Figure 3D,E). In contrast, when FH of BC in 1st degree relatives was further restricted to affected relatives only, 7.2% of women were reclassified, with 4.8% being reclassified to a higher risk category (Figure 3F).

### 3.4. Impact of Specific Groups of Questionnaire Risk Factors on Risk Classification

**Figure 4:**
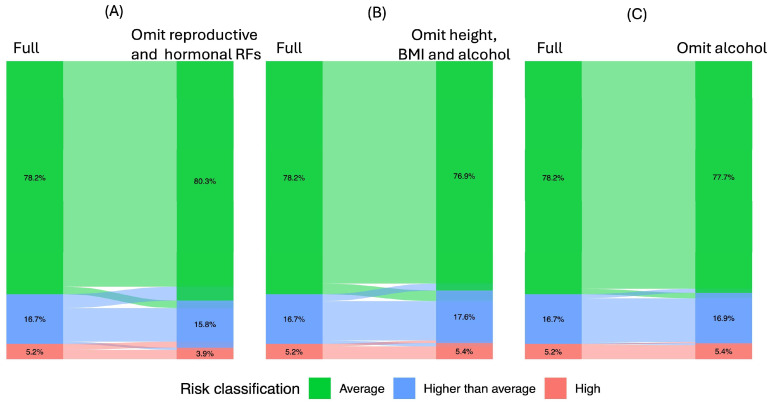
Risk reclassification when omitting different sets of questionnaire-based risk factors from the full model. (**A**) Omitting reproductive and hormonal risk factors (including age at menarche, age at menopause, parity, age at first live birth, oral contraceptive use and MHT use); (**B**) omitting lifestyle risk factors (including BMI, height and alcohol intake); (**C**) omitting alcohol intake. The flows illustrate transitions in individual risk classification from the full model (**left**) to the reduced model with specified risk factors omitted (**right**), with the width of each flow representing the proportion of individuals reclassified between risk categories.

When considering specific groups of QRFs, reproductive and hormonal risk factors (age at menarche, age at menopause, parity, age at first live birth, oral contraceptive use and MHT use) had a greater influence on risk classification (reclassification = 9.9%, *p* = 0.012) compared to body mass index (BMI), height and alcohol intake (reclassification = 7.9%, *p* = 0.43) (Figure 4, Table 3). Omitting alcohol intake alone from the full model resulted in an overall reclassification rate of 4.2% (*p* = 0.88, Figure 4, Table 3).

### 3.5. Supplementing Data Already Collected During Screening

**Figure 5:**
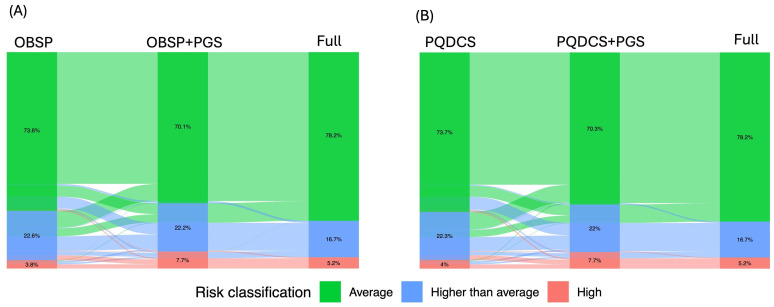
Risk reclassification when using supplementing data collected during the current breast cancer screening programs. (**A**) Full model versus using OBSP risk factor sets versus OBSP risk factor sets plus PGS; (**B**) full model versus PQDCS risk factor sets versus PQDCS risk factor sets plus PGS. Information on affected relatives only in the first- degree relatives was collected in both OBSP and PQDCS breast cancer screening programs.

Using PQDCS risk factors routinely collected in screening programs (Table A2), 23.4% of women were reclassified into different risk categories. Of these, 57.6% were moved to a higher risk category, while 42.4% to a lower risk category. When the PGS was added to the PQDCS risk factors, the reclassification compared to the full model dropped to 12.4%, with 91.6% of reclassified women shifted to a higher risk category and 8.4% to a lower risk category (Table 4). A similar pattern was observed using the OBSP risk factor set (Figure 5 and Table 4). It should be noted that the observed residual reclassification to a higher risk category relative to the full model was primarily driven by the fact that these programs only collect information on affected family members, without accounting for unaffected relatives.

### 3.6. Risk Reclassification for Women Meeting the Current Annual Mammogram Screening Criteria in Ontario

**Figure 6:**
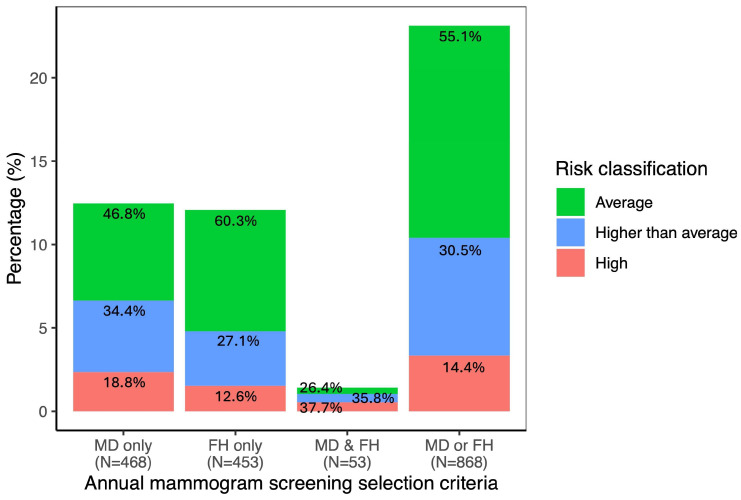
Risk reclassification under the full model for women who met the annual mammogram screening criteria in Ontario. (MD: women with BI-RADS^®^ density D; FH: women met the cancer family history criteria).

Women eligible for annual mammography screening in Ontario are considered at “higher-than-average risk”. In our cohort, 468 women (12.5% of the cohort) met the current Ontario criteria for annual mammographic screening based on BI-RADS^®^ density D (Figure 5). However, under the full model, 219 (46.8%) of these women would be reclassified as average-risk (5.8% of the cohort), while 88 (18.8%) would be reclassified as high-risk (2.3% of the cohort). Similarly, 454 women (12.1% of the cohort) would have been eligible for annual mammographic screening based on the FH criteria (see Materials and Methods Section 2.2). When using the full model, 273 (60.3%) of these women would be reclassified as average-risk (7.3% of the cohort), and 57 (12.6%) as high-risk (1.5% of the cohort) (Figure 6). In total, 868 women (23.1% of the cohort) met the annual mammogram screening eligibility based on either BI-RADS^®^ density D, FH or both. Using the full model, 478 (55.1%) of these women would be reclassified as average-risk (12.7% of the cohort), and 125 (14.4%) as high-risk (3.3% of the cohort) (Figure 6).

## 4. Discussion

Using a detailed quantification of risk reclassification flows, this study is the first to evaluate how individual risk factors contribute to BC risk estimates in a manner that can directly impact risk stratification and inform breast cancer screening programs. Our study has notably shown the substantial impact of PGS on individual-level risk stratification, consistent with findings from other established breast cancer risk models, including Breast Cancer Screening Consortium (BCSC) [12,40], Mammorisk^®^ [40] and iCARE [41]. Our findings have also shown that omitting PGS, FH, or MD led to a greater proportion of reclassification among women aged 40–49 years compared to those aged 50–70 years. In contrast, omission of QRFs had a more pronounced effect in women aged 50–70 years. This is consistent with previous evidence showing that PGS and FH provide stronger relative discrimination for earlier-onset BC [38,42,43]. These findings support the concept that risk-based screening strategies may benefit from age-adapted implementation, in which the relative emphasis on different classes of risk factors is balanced according to age-specific risk architecture and feasibility of data collection. Importantly, we demonstrate that collecting FH of BC only is sufficient for effective risk stratification, with limited added value from collecting FH of other cancers, such as ovarian, pancreas or prostate cancer. However, it is essential to collect both affected and unaffected first- and second-degree relatives. Collecting information only on affected relatives, as is done in some existing screening programs, led to a substantial overestimation of risk, with over 97% of reclassified women being shifted into a higher risk category. Together, these findings highlight the importance of a multifactorial approach to BC risk assessment and indicate that omitting any risk factor categories may result in an important proportion of women not receiving appropriate screening recommendations and preventive measures.

More specifically, omitting PGS, FH, or MD resulted in significant differences in the distribution of women across risk categories compared to the full model. Among women who were reclassified after PGS or FH was excluded, a greater proportion moved to a lower risk category, highlighting the critical role of both PGS and FH in identifying women at elevated BC risk. In contrast, omitting MD or QRFs led to a more balanced reclassification, with similar proportions of women moving to a lower or a higher risk category. Notably, the exclusion of MD had a substantial impact on individual risk stratification, underscoring the importance of women being informed about their breast density and its clinical relevance for breast cancer risk assessment and screening decisions. The impact of excluding PGS, FH, or MD was more pronounced among women aged 40–49 years than among those aged 50–70 years (Figure 2). This may be partly explained by the study’s recruitment criteria, which required prior mammography, likely enriching the younger age group for women with cancer FH and thus a higher baseline risk [23]. Consequently, the observed differences in risk impact and reclassification in this younger age group should be interpreted with caution, as they may partly reflect selection effects rather than true age-specific differences in a population-representative screening cohort. The effectiveness of PGS in identifying higher-risk individuals in the 40–49 age group aligns with findings from a previous validation study conducted in the UK Biobank [35].

Among QRFs, reproductive and hormonal factors contributed most to risk reclassification, underscoring their important role in BC risk. Although lifestyle factors such as BMI and alcohol intake had limited impact on individual risk classification, they remain important modifiable risk factors at the population level and present targets for prevention strategies [44,45]. In addition, these factors are typically based on objective or routinely collected clinical data and are relatively easy to ascertain. Including these factors in risk assessments may therefore enhance feasibility and also empower women to make informed decisions about their breast health and adopt lifestyle changes that can benefit their overall well-being.

Results from our recent PERSPECTIVE I&I project have illustrated the significant challenge and resource-intensive nature of collecting comprehensive and accurate risk factor information [23]. This is particularly true for the collection of a comprehensive first- and second-degree FH of breast, ovarian, pancreatic and prostate cancer, which represented a significant burden for participants and required extensive verification [23,25], emphasizing the need to reduce the burden associated with risk factor data collection in order to maximize participation in risk assessment and to help facilitate the implementation of a risk-based screening approach at a population level [23]. To this end, the current study has demonstrated that restricting FH collection to first- and second-degree relatives’ BC status is sufficient for effective BC risk stratification. Importantly, our analyses also highlight the critical role of including information on both affected and unaffected relatives. As observed in the FH scenario that includes BC in first- and second-degree relatives, restricting to only affected family members results in significantly higher predicted risks, with 8.2% of women being reclassified in a higher risk category when unaffected relatives are not considered. This latter observation has important implications for the implementation of a risk-based screening approach, as it could result in unnecessary intensification of screening for a substantial proportion of women who would otherwise be at lower risk of the disease. Taken together, these findings illustrate a key trade-off between predictive contribution, measurement robustness, and feasibility. While detailed FH of BC in both affected and unaffected first- and second-degree relatives is challenging and resource-intensive to collect, it has a substantial impact on risk classification and should be prioritized. In contrast, other FH components (e.g., non-breast cancers) that require similar effort but contribute minimally to risk discrimination may reasonably be omitted in implementation settings. Future work should continue to evaluate how best to balance predictive accuracy with practicality, particularly by considering the inclusion of variables that are both robustly measured and feasible to collect at scale.

Our analyses clearly demonstrate the impact of supplementing the existing risk factor data currently collected in the population breast screening programs in Ontario (OBSP) and Quebec (PQDCS). In both provinces, using only the set of risk factors already collected resulted in nearly one in four women (approximately 23% of the cohort) being differentially classified compared to the full model. Incorporating the PGS reduced the proportion of reclassified women to around 13%, although overestimation of risk was still observed compared to the full model. This overestimation is largely driven by the fact that these programs collect information only on affected family members, without accounting for unaffected relatives, which, as discussed above, leads to overprediction. This pattern of overestimation may have important implications for implementation, as it could lead to a larger proportion of women being recommended for more intensive screening than necessary, with potential consequences including increased healthcare burden, unnecessary investigations, and possible overdiagnosis. These findings highlight the importance of collecting family history on both affected and unaffected relatives to ensure accurate and clinically appropriate risk stratification.

The importance of a comprehensive multifactorial risk assessment approach to ensure appropriate screening and preventative measures was further underscored by comparison to the current criteria used by OBSP to identify women at higher-than-average risk. Under existing eligibility criteria, having breast density classified as BI-RADS^®^ “D” at the time of screening, or meeting specific cancer FH criteria (see Materials and Methods Section 2.2), nearly one quarter of the study cohort would qualify for annual mammography. However, when assessed using multifactorial risk assessment, over half of these women (55.1%) would be reclassified as average-risk, while 14.4% would be reclassified as higher risk and may benefit from more intensive screening by adding magnetic resonance imaging (MRI). The substantial reclassification observed relative to current OBSP criteria also has important health system and ethical implications. Risk-stratified screening offers the potential to better balance the benefits and harms of mammography across different risk groups, for example, by reducing screening intensity among women at lower risk while maintaining or enhancing surveillance for those at higher risk. However, such a shift raises important considerations regarding acceptability, equity, and communication. Evidence from population-based survey studies [46] suggests that willingness to adopt risk-stratified screening is strongly influenced by individual risk perception and understanding. As such, communication strategies must carefully consider how risk information is conveyed to support informed decision-making and public trust. Framing risk-stratified screening as an approach to optimize the balance between benefits and harms across risk strata may improve acceptability, but further work is needed to evaluate its implementation in practice. It should be kept in mind that MD, in addition to being an important risk factor in itself, can influence the detection of breast cancers by standard mammography in women with dense breasts. Note that it has been shown that annual mammography screening for women with dense breasts reduces interval cancers compared to biennial screening, thereby highlighting the benefits of annual screening for these women irrespective of risk [47]. Moreover, a recent controlled randomized trial, performed in women aged 50–70 years old with dense breasts and a normal mammogram, has shown that abbreviated MRI led to increased cancer detection rates compared to standard care, demonstrating that MRI, and other supplemental imaging techniques, delivered in the context of screening programs could be effective for early detection of cancer in women with dense breasts regardless of their assessed risk [48].

Our results are consistent with previous studies demonstrating the added value of multifactorial risk assessment in the context of population breast cancer screening and the contribution of PGS to model performance [35,49,50]. Similar to earlier work, we observed that the addition of QRFs, MD and/or PGS results in more women being classified as at high risk [51]. However, unlike those studies, one of the strengths of this study is the quantification of reclassification flows, which reveals substantial individual-level reclassification, even when the overall distribution of women across risk categories remains relatively stable. This approach provides a more nuanced understanding of how each risk factor contributes to risk classification and underscores the importance of risk factors such as QRF that may not significantly alter the overall population-level risk category proportions but impact substantially individual risk categorization. Another strength is the large sample size, comprising 3753 women with comprehensive risk factor information, particularly MD and detailed FH, both of which are not available in previous validation studies using the UK Biobank data [35].

However, certain limitations of our study may affect the generalizability of the findings. First, we acknowledge that only the BOADICEA model was used to determine risk estimates. BOADICEA was chosen as the risk prediction tool in this study because it is the only model capable of explicitly evaluating the effects of different levels and types of family history, and it has been well validated in multiple prospective cohorts [33,34,35,36]. Another limitation pertains to risk reclassifications among younger women. Recruitment requirements and convenience sampling, especially in Quebec, resulted in a higher proportion of women aged 40–49 years who had undergone opportunistic mammographic screening. This likely introduced self-selection bias, as these women were more likely to have known risk factors; for example, a higher proportion of participants with dense breasts or a family history of cancer was observed in the study. Consequently, the prevalence of higher-risk individuals in this age group may have been overestimated, potentially inflating reclassification rates compared to what would be expected in the general population [23]. Based on the current limited acceptability of reducing screening frequencies for individuals at low risk [46,52], the PERSPECTIVE I&I study design did not include a low-risk group. Additionally, our study design did not include analysis of pathogenic variants in high- and moderate-penetrance BC susceptibility genes. Although these variants are important for identifying individuals at very high risk of BC, their contribution to population-level risk stratification is relatively limited [22,50,53]. Another limitation is the demographic composition of our cohort, with 89.6% of participants being white women. Future research should include more ethnically diverse populations to ensure broader applicability. This will be facilitated by the release of the new multi-ethnicity version of the BOADICEA model [54]. Lastly, we acknowledge that the risk estimates provided in the current study were not validated against real cancer outcomes, as breast cancer incidence data were not available due to the design of the study. However, comparison with the current eligibility criteria used to identify women at higher-than-average risk underscores the clinical relevance of multifactorial risk assessment to optimize screening strategies. Future prospective follow-up will be needed to assess how reclassification impacts health outcomes.

Importantly, the implications of multifactorial risk assessment for screening strategies must consider screening intervals, which are informed by the sojourn time, defined as the preclinical period during which cancer is asymptomatic but detectable by screening. Bhatt et al. (2024) show that the duration of this period does not vary substantially across risk groups; however, the age at which women enter this screen-detectable phase differs, with high-risk women entering earlier than low-risk women [4]. Because high-risk women have a greater underlying risk of BC, initiating screening at a younger age and conducting it more frequently increases the chance of detecting cancer early. In contrast, for low-risk women, reducing screening burden is more effectively achieved by delaying the age of screening initiation rather than by extending the interval between screens.

An additional consideration in risk-stratified screening is the potential for overdiagnosis. As screening intensity is increased in higher-risk women and reduced in lower-risk women, there is corresponding potential for both increases and decreases in overdiagnosis across groups. The overall impact, therefore, cannot be inferred from individual subgroups and must be evaluated at the program level. Moreover, it is important to assess risk-based screening in terms of its overall benefit–harm balance, particularly the trade-off between reductions in BC mortality and increases in overdiagnosis. In this context, cost-effectiveness studies suggest that risk-stratified approaches can improve the overall benefit–harm balance and are cost-effective compared with uniform screening strategies [55,56].

## 5. Conclusions

Multifactorial risk assessment enhances BC risk stratification and supports a more personalized approach to screening and prevention compared to current programs using age, FH or MD alone. Overall, the findings of this study highlight the importance of a comprehensive approach to personalized risk assessment in order to tailor breast screening to individual risk and demonstrate that effective risk prediction can be achieved using a focused set of variables, including PGS, MD, QRFs and FH of BC, only in both affected and unaffected family members. This approach reduces data collection burden without compromising model performance. Notably, exclusion of the PGS led to the highest reclassification and addition of the PGS to the data routinely collected as part of the provincial screening programs closely aligned with the risk stratification of the full model. Ultimately, decisions about which risk factors to include in screening programs will depend on the trade-offs between the additional costs and resources required for risk factor data collection, the benefits and harms of screening, and the benefits of accurate risk classification, while ensuring equitable access. This study provides essential evidence to inform those decisions and guide the implementation of personalized risk-based screening strategies.

## Figures and Tables

**Table 1:** Risk reclassification using different sets of risk factors compared to the full model using: (A) the entire dataset; (B) age group 40–49 years; and (C) age group 50–70 years.

(A) Using the Entire Dataset												
Full Model	Average (N = 2934)			Higher-Than-Average (N = 625)			High (N = 194)			*p*-Value	N.Higher (%)	N.Lower (%)
Models Compared	Average	Higher-Than-Average	High	Average	Higher-Than-Average	High	Average	Higher-Than-Average	High			
Omitting one key category of risk factors												
omit PGS	2756	176 (6%)	2 (0.1%)	359 (57.4%)	245	21 (3.4%)	38 (19.6%)	103 (53.1%)	53	1.5 × 10^−15^	199 (5.3%)	500 (13.3%)
omit FH	2840	94 (3.2%)	0 (0%)	200 (32%)	412	13 (2.1%)	16 (8.2%)	63 (32.5%)	115	9.0 × 10^−5^	107 (2.9%)	279 (7.4%)
omit MD	2739	190 (6.5%)	5 (0.2%)	149 (23.8%)	444	32 (5.1%)	0 (0%)	78 (40.2%)	116	4.4 × 10^−3^	227 (6%)	227 (6%)
omit QRF	2758	176 (6%)	0 (0%)	184 (29.4%)	398	43 (6.9%)	4 (2.1%)	73 (37.6%)	117	0.16	219 (5.8%)	261 (7%)
**(B) In Age Group 40–49 Years**												
**Full Model**	**Average (N = 333)**			**Higher-Than-Average (N = 103)**			**High (N = 69)**			***p*-Value**	**N.Higher** **(%)**	**N.Lower** **(%)**
**Models Compared**	**Average**	**Higher-Than-Average**	**High**	**Average**	**Higher-Than-Average**	**High**	**Average**	**Higher-Than-Average**	**High**			
omit PGS	289	42 (12.6%)	2 (0.6%)	46 (44.7%)	49	8 (7.8%)	8 (11.6%)	30 (43.5%)	31	0.013	52 (10.3%)	84 (16.6%)
omit FH	327	6 (1.8%)	0 (0%)	47 (45.6%)	56	0 (0%)	10 (14.5%)	23 (33.3%)	36	3.9 × 10^−4^	6 (1.2%)	80 (15.8%)
omit MD	309	23 (6.9%)	1 (0.3%)	30 (29.1%)	63	10 (9.7%)	0 (0%)	22 (31.9%)	47	0.57	34 (6.7%)	52 (10.3%)
omit QRF	310	23 (66.9%)	0 (0%)	14 (13.6%)	75	14 (13.6%)	0 (0%)	12 (17.4%)	57	0.83	37 (7.3%)	26 (5.1%)
**(C) In Age Group 50–70 Years**												
**Full Model**	**Average (N = 2601)**			**Higher-Than-Average (N = 522)**			**High (N = 125)**			***p*-Value**	**N.Higher** **(%)**	**N.Lower** **(%)**
**Models Compared**	**Average**	**Higher-Than-Average**	**High**	**Average**	**Higher-Than-Average**	**High**	**Average**	**Higher-Than-Average**	**High**			
omit PGS	2467	134 (5.2%)	0 (0%)	313 (60.0%)	196	13 (2.5%)	30 (24.0%)	73 (58.4%)	22	8.5 × 10^−17^	147 (4.5%)	416 (12.8%)
omit FH	2513	88 (3.4%)	0 (0%)	153 (29.3%)	356	13 (2.5%)	6 (4.8%)	40 (32%)	79	0.025	101 (3.1%)	199 (6.1%)
omit MD	2430	167 (6.4%)	4 (0.2%)	119 (22.8%)	381	22 (4.2%)	0 (0%)	56 (44.8%)	69	5.0 × 10^−3^	193 (5.9%)	175 (5.4%)
omit QRF	2448	153 (5.9%)	0 (0%)	170 (32.6%)	323	29 (5.6%)	4 (3.2%)	61 (48.8%)	60	0.042	182 (5.6%)	235 (7.2%)

The numbers in brackets represent the proportion of reclassification rates within each category relative to the full model. *p*-values were calculated using the chi-square test to determine whether the overall proportions of each risk category significantly differed from those in the full model. N.higher indicates the number of women reclassified to a higher risk category relative to the full model, with the proportion among the total women shown in brackets. N.lower indicates the number of women reclassified to a lower risk category relative to the full model, with the proportion among the total women shown in brackets.

**Table 2:** Risk reclassification using different sets of family history information compared to the full model.

Full Model	Average (N = 2934)			Higher-Than-Average (N = 625)			High (N = 194)			*p*-Value	N.Higher (%)	N.Lower (%)
Models Compared	Average	Higher-Than- Average	High	Average	Higher-Than- Average	High	Average	Higher-Than- Average	High			
Full model, but considering the following in the family history												
1st & 2nd degree relatives in BC & OC	2934	0 (0%)	0 (0%)	7 (1.1%)	618	0 (0%)	1 (0.5%)	4 (2.1%)	189	0.96	0 (0%)	12 (0.3%)
1st & 2nd degree relatives in BC	2934	0 (0%)	0 (0%)	11 (1.8%)	614	0 (0%)	1 (0.5%)	7 (3.6%)	186	0.90	0 (0%)	19 (0.5%)
1st & 2nd degree relatives in BC without considering unaffected relatives	2713	220 (7.5%)	1 (0.03%)	5 (0.8%)	535	85 (13.6%)	0 (0%)	3 (1.5%)	191	1.8 × 10^−8^	306 (8.2%)	8 (0.2%)
1st degree relatives in BC & OC & PAN & PRO	2862	72 (2.5%)	0 (0%)	83 (13.3%)	518	24 (3.8%)	4 (2.1%)	30 (15.5%)	160	0.85	96 (2.6%)	117 (3.1%)
1st degree relatives in BC	2867	67 (2.3%)	0 (0%)	84 (13.4%)	517	24 (3.8%)	5 (2.6%)	31 (16%)	158	0.76	91 (2.4%)	120 (3.2%)
1st degree relatives in BC without considering unaffected relatives	2793	141 (4.8%)	0 (0%)	60 (9.6%)	526	39 (6.2%)	3 (1.5%)	26 (13.4%)	165	0.09	180 (4.8%)	89 (2.4%)

See Table 1 legend for detailed description. BC: breast cancer; OC: ovarian cancer; PAN: pancreatic cancer; PRO: prostate cancer.

**Table 3:** Risk reclassification, omitting different sets of questionnaire risk factors compared to the full model.

Full Model	Average (N = 2934)			Higher-Than-Average (N = 625)			High (N = 194)			*p*-Value	N.Higher (%)	N.Lower (%)
Model Compared	Average	Higher-Than-Average	High	Average	Higher-Than-Average	High	Average	Higher-Than-Average	High			
Full model but omitting the following sets of questionnaire risk factors												
Omit reproductive & hormonal RFs	2840	94 (3.2%)	0 (0%)	175 (28%)	423	27 (4.3%)	0 (0%)	75 (38.7%)	119	0.012	121 (3.2%)	250 (6.7%)
Omit height, BMI and alcohol	2800	134 (4.6%)	0 (0%)	87 (13.9%)	496	42 (6.7%)	0 (0%)	32 (16.5%)	162	0.43	176 (4.7%)	119 (3.2%)
Omit alcohol	2866	68 (2.3%)	0 (0%)	51 (8.2%)	552	22 (3.5%)	0 (0%)	15 (7.7%)	179	0.88	90 (2.4%)	66 (1.8%)

See Table 1 legend for detailed description.

**Table 4:** Risk reclassification when using risk factors routinely collected in Quebec (PQDCS) and Ontario (OBSP) screening programs compared to the full model.

Full Model	Average (N = 2934)			Higher-Than-Average (N = 625)			High (N = 194)			*p*-Value	N.Higher (%)	N.Lower (%)
Model Compared	Average	Higher-Than- Average	High	Average	Higher-Than- Average	High	Average	Higher-Than- Average	High			
Supplementing data already collected during screening												
PQDCS	2484	430 (14.7%)	20 (0.7%)	253 (40.5%)	315	57 (9.1%)	28 (14.4%)	92 (47.4%)	74	1.2 × 10^−9^	507 (13.5%)	373 (9.9%)
PQDCS + PGS	2613	320 (10.9%)	1 (0.03%)	25 (4.0%)	493	107 (17.1%)	0 (0%)	14 (7.2%)	180	3.2 × 10^−14^	428 (11.4%)	39 (1%)
OBSP	2488	430 (14.7%)	16 (0.5%)	246 (39.4%)	327	52 (8.3%)	28 (14.4%)	92 (47.4%)	74	5.4 × 10^−11^	498 (13.3%)	366 (9.8%)
OBSP + PGS	2596	335 (11.4%)	3 (0.1%)	36 (5.8%)	479	110 (17.6%)	0 (0%)	18 (9.3%)	176	1.0 × 10^−14^	448 (11.9%)	54 (1.4%)

See Table 1 legend for detailed description. PQDCS: Programme québécois de dépistage du cancer du sein; OBSP: Ontario Breast Screening Program.

## Data Availability

Parts of the material supporting this article are based on data and information provided by Ontario Health (Cancer Care Ontario). Ontario Health is prohibited from making the data used in this research publicly accessible if it includes potentially identifiable personal health information and/or personal information as defined in Ontario law, specifically the Personal Health Information Protection Act (PHIPA) and the Freedom of Information and Protection of Privacy Act (FIPPA). Upon request, data de-identified to a level suitable for public release may be provided. A subset of the Quebec participants has consented to sharing their data in the context of future research. De-identified data from these participants are available upon request to the principal investigator of the PERSPECTIVE I&I project (jacques.simard@crchudequebec.ulaval.ca). For the other subset of Quebec participants, data cannot be shared due to consent form constraints. No personally identifiable information will be shared.

## Abbreviations

The following abbreviations are used in this manuscript:

BC	Breast cancer
QRF	Questionnaire-based risk factor
PGS	Polygenic score
MD	Mammographic density
FH	Family history
MRI	Magnetic resonance imaging
BOADICEA	Breast and Ovarian Analysis of Disease Incidence and Carrier Estimation Algorithm
SNP	Single-nucleotide polymorphism
PERSPECTIVE I&I	Personalized Risk Assessment for Prevention and Early Detection of Breast Cancer: Integration and Implementation
OC	Ovarian cancer
PAN	Pancreatic cancer
PRO	Prostate cancer
MHT	Menopausal hormone therapy
OBSP	Ontario Breast Screening Program
PQDCS	*Programme québécois de dépistage du cancer du sein*
RR	Relative risk
BMI	Body mass index

## Appendix A

**Table A1:** A summary of the participant characteristics in the PERSPECTIVE cohort.

	Number	%
Number of women	3753	
Study Center		
Ontario	2111	56.2
Quebec	1642	43.8
Age at risk assessment		
[40, 45)	223	5.9
[45, 50)	282	7.5
[50, 55)	602	16.0
[55, 60)	928	24.7
[60, 65)	957	25.5
[65, 70)	752	20.0
≥70	9	0.2
median (IQR), years	59 (54–64)	
Ashkenazi	101	2.7
Age at menarche		
<11	198	5.5
11	479	13.2
12	1004	27.6
13	1011	27.8
14	557	15.3
15	219	6.0
≥16	164	4.5
Missing	121	
Parity		
0	803	21.5
1	564	15.1
2	1624	43.4
≥3	749	20.0
Missing	13	
Age at first live birth (among parous women)		
<20	100	3.4
[20, 25)	548	18.7
[25, 30)	1178	40.1
≥30	1109	37.8
Missing	2	
Use of oral contraceptive		
Current	177	4.8
Former	3145	85.0
Never	377	10.2
Missing	54	
Duration of oral contraceptive		
Never or <1	646	17.7
[1, 4]	603	16.5
[5, 9]	708	19.4
[1–14]	725	19.9
≥15	970	26.6
Missing	101	
Use of hormonal replacement treatment		
Current estrogen-only type	272	9.6
Current other type	304	10.8
Former	358	12.7
Never	1889	66.9
Missing	29	
Body Mass Index (kg/m^2^)		
<18.5	60	1.6
[18.5, 25)	1673	44.7
[25, 30)	1130	30.2
≥30	876	23.4
Missing	14	
Height (cm)		
<153	209	5.6
[153, 160)	711	19.0
[160, 166)	1614	43.1
[166, 173)	842	22.5
≥173	373	9.9
Missing	4	
Alcohol consumption (g/day)		
<5	1672	47.1
[5, 15)	1167	32.9
[15, 25)	340	9.6
[25, 35)	229	6.5
[35, 45)	91	2.6
≥45	63	1.8
Missing	191	
Menopausal status		
Pre-menopausal	895	23.9
Post-menopausal	2852	76.1
Missing	6	
Age at menopause (among post-menopausal women)		
<40	185	7.1
[40, 45)	204	7.8
[45, 50)	508	19.5
[50, 55)	1239	47.7
≥55	464	17.8
Missing	252	
BIRADS		
1 or a	282	9.1
2 or b	1318	42.5
3 or c	1031	33.3
4 or d	468	15.1
Missing	654	
Ethnicity		
Black	35	0.9
East Asian	92	2.5
Indigenous	43	1.1
Latin American/Hispanic	41	1.1
Arab	5	0.1
West Asian	11	0.3
South Asian	24	0.6
Southeast Asian	30	0.8
White	3361	89.6
Mixed	38	1.0
unspecified	73	1.9
PGS		
[−3.831, −1.222)	374	10.0
[−1.222, 0.141)	1500	40.0
[0.141, 1.478)	1501	40.0
[1.478, 3.805]	375	10.0
mean	0.138	
sd	1.045	
median	0.141	
missing	3	
Number of women with FH of BC in		
both 1st and 2nd degree relatives	338	9.0
1st degree relatives only	569	15.2
2nd degree relatives only	881	23.5
none in 1st or 2nd degree relatives	1965	52.4
Number of women with FH of BC or OC in		
both 1st and 2nd degree relatives	395	10.5
1st degree relatives only	620	16.5
2nd degree relatives only	955	25.4
none in 1st or 2nd degree relatives	1783	47.5
Number of women with FH of BC, OC, PAN or PRO in		
both 1st and 2nd degree relatives	704	18.8
1st degree relatives only	718	19.1
2nd degree relatives only	1038	27.7
none in 1st or 2nd degree relatives	1293	34.5

**Table A2:** List of risk factors considered in the full model and in the Ontario Breast Screening Program (OBSP) and the Programme québécois de dépistage du cancer du sein (PQDCS).

	Full Model	OBSP	PQDCS
PGS	X		
MD	X	X	X
FH of BC in 1st degree relatives	X		
FH of BC in 1st degree relatives with affected relatives only		X	X
FH of OC in 1st degree relatives	X		
FH of OC in 1st degree relatives with affected relatives only		X	
FH of BC and OC in 2nd degree relatives	X		
FH of PRO and PAN cancer in 1st and 2nd degree relatives	X		
Height	X		X
BMI	X		X
Parity	X	X	X
Age at recruitment	X	X	X
Age at first live childbirth	X	X	X
Age at menarche	X	X	
Age at menopause	X	X	X
Oral contraceptive use	X		
MHT use	X	X	X
Alcohol intake	X		
